# Commonly used genomic arrays may lose information due to imperfect coverage of discovered variants for autism spectrum disorder

**DOI:** 10.1186/s11689-024-09571-8

**Published:** 2024-09-12

**Authors:** Michael Yao, Jason Daniels, Luke Grosvenor, Valerie Morrill, Jason I. Feinberg, Kelly M. Bakulski, Joseph Piven, Heather C. Hazlett, Mark D. Shen, Craig Newschaffer, Kristen Lyall, Rebecca J. Schmidt, Irva Hertz-Picciotto, Lisa A. Croen, M. Daniele Fallin, Christine Ladd-Acosta, Heather Volk, Kelly Benke

**Affiliations:** 1 Mental Health, Johns Hopkins Bloomberg School of Public Health, Baltimore, MD USA; 2 Epidemiology, Johns Hopkins Bloomberg School of Public Health, Baltimore, MD USA; 3 Wendy Klag Center for Autism and Developmental Disabilities, JHSPH, Baltimore, MD USA; 4 Department of Epidemiology, School of Public Health, University of Michigan, Ann Arbor, MI USA; 5 Department of Psychiatry, University of North Carolina, North Carolina, Chapel Hill, 27599 USA; 6 Carolina Institute for Developmental Disabilities, Chapel Hill, NC 27599 USA; 7 7AJ Drexel Autism Institute, Drexel University, 3020 Market St, Suite 560, Philadelphia, PA 19104 USA; 8 College of Health and Human Development, Penn State, University Park, PA 16802 USA; 9 Department of Public Health Sciences, University of California, Davis, CA 95616 USA; 10 UC Davis MIND (Medical Investigations of Neurodevelopmental Disorders) Institute, Sacramento, CA 95817 USA; 11 Autism Research Program, Kaiser Permanente Division of Research, 2000 Broadway, Oakland, CA 94612 USA; 12 Rollins School of Public Health, Emory University, 1518 Clifton Rd, Suite 8011, Atlanta, GA 30355 USA

**Keywords:** Polygenic scores (PGS), Autism spectrum disorder (ASD), Information Loss

## Abstract

**Background:**

Common genetic variation has been shown to account for a large proportion of ASD heritability. Polygenic scores generated for autism spectrum disorder (ASD-PGS) using the most recent discovery data, however, explain less variance than expected, despite reporting significant associations with ASD and other ASD-related traits. Here, we investigate the extent to which information loss on the target study genome-wide microarray weakens the predictive power of the ASD-PGS.

**Methods:**

We studied genotype data from three cohorts of individuals with high familial liability for ASD: The Early Autism Risk Longitudinal Investigation (EARLI), Markers of Autism Risk in Babies-Learning Early Signs (MARBLES), and the Infant Brain Imaging Study (IBIS), and one population-based sample, Study to Explore Early Development Phase I (SEED I). Individuals were genotyped on different microarrays ranging from 1 to 5 million sites. Coverage of the top 88 genome-wide suggestive variants implicated in the discovery was evaluated in all four studies before quality control (QC), after QC, and after imputation. We then created a novel method to assess coverage on the resulting ASD-PGS by correlating a PGS informed by a comprehensive list of variants to a PGS informed with only the available variants.

**Results:**

Prior to imputations, None of the four cohorts directly or indirectly covered all 88 variants among the measured genotype data. After imputation, the two cohorts genotyped on 5-million arrays reached full coverage. Analysis of our novel metric showed generally high genome-wide coverage across all four studies, but a greater number of SNPs informing the ASD-PGS did not result in improved coverage according to our metric.

Limitations.

The studies we analyzed contained modest sample sizes. Our analyses included microarrays with more than 1-million sites, so smaller arrays such as Global Diversity and the PsychArray were not included. Our PGS metric for ASD is only generalizable to samples of European ancestries, though the coverage metric can be computed for traits that have sufficiently large-sized discovery findings in other ancestries.

**Conclusions:**

We show that commonly used genotyping microarrays have incomplete coverage for common ASD variants, and imputation cannot always recover lost information. Our novel metric provides an intuitive approach to reporting information loss in PGS and an alternative to reporting the total number of SNPs included in the PGS. While applied only to ASD here, this metric can easily be used with other traits.

**Supplementary Information:**

The online version contains supplementary material available at 10.1186/s11689-024-09571-8.

## Background

PolyGenic Scores (PGS) are potentially useful tools for research and prediction [1, 2], but require additional development before their utility can be fully realized [3]. PGS are weighted sums of risk alleles that are computed using genotype data from individuals in a select target sample. The list of genetic variants to be summed and attendant effect sizes that serve as weights are identified in large-scale, genome-wide discovery studies. PGS represent an individual’s genetic loading for a given trait, and although they are not expected to be sufficiently predictive for psychiatric and mental health outcomes on their own [4], they are likely to serve in the future as an essential component of risk modeling that guides clinical decision making for preventive strategies or post-diagnostic treatment.

Several factors limit the potential utility of the PGS by either directly or indirectly influencing the extent to which the PGS captures the true genetic susceptibility. Some of these factors are not easily addressed. Lack of generalizability between discovery and target samples, for example, will require data collection in diverse ancestries around the world [5, 6]. Also, the sample size or phenotypic measurement employed in current discovery studies may result in power loss reflecting incompleteness of variant identification and imprecision of effect sizes to serve as weights in the PGS [7]. Regardless of the completeness of discovery, when applying discovery results to target genotypes, the incomplete overlap of the discovery variant list and target genotyping array data can also lead to information loss when computing PGS. Specifically, the genotyping array used in the target sample is likely to lack some portion of the index discovery variants, and this will differ by array. Even if correlated, proxy variants are available as substitutes for the index variant, there is still some expected loss of PGS signal. It is often assumed that genotype imputation can recoup the lost information from unrepresented polymorphisms [8]. However, if no index or proxy genetic variants can be imputed with high quality, information loss will occur. Collectively, this lack of discovery representation in the genetic variants in the target sample could lead to a lower observed variance explained compared to the initial PGS R^2^ metric often reported as part of the large discovery GWAS effort [9].

PGS for numerous psychiatric traits have been developed and have both global and unique considerations for their use and development. This project focuses on a PGS for Autism Spectrum Disorder (ASD). ASD is a neurodevelopmental disability characterized by social interaction and social communication deficits, and restrictive, repetitive patterns in behaviors, interests, or activities [10]. The heritability for common genetic variation is substantial and was estimated to be about 50% [11]. A PGS for ASD (ASD-PGS) could assist in the early detection of infants or toddlers displaying early symptoms. ASD-PGS, if accurate in the prediction of later abilities and challenges among diagnosed children, could also be used to inform parents and guide decisions about more specific and individualized supports and services.

To inform the ASD-PGS, the most recent discovery effort by Grove et al. (2019) identified common genetic susceptibility variants predisposing to ASD and was accomplished via genome-wide association study (GWAS) in combined samples from the Psychiatric Genomic Consortia (PGC) and the Danish Lundbeck Foundation Initiative for Integrative Psychiatric Research (iPSYCH) studies [12]. This scan successfully identified three independent loci and then attempted to replicate the findings for the top 88 variants using meta-analysis of five follow up studies conducted in Northern European populations. In addition to the top 88 GWAS hits, the Grove et al. study evaluated the predictive ability of ASD-PGS. While confirming that common genetic prediction is not currently clinically useful [1], the ASD-PGS was observed to explain 2.8% of variance in the trait [12], the SNP-based heritability, representing the potential of polygenic prediction with sufficient discovery sample size, was estimated at 11% [13].

A number of recent studies have used the Grove et al. discovery information to derive ASD-PGS and found statistically significant associations with ASD [14–17] and other ASD-related traits [18–20]. However, when reported, variance explained is well below the value estimated in Grove et al. (2019) and measures of the strength of association are modest or null. The failure to achieve the full predictive power of the ASD-PGS can be attributed to small target sample size and lack of generalizability of the target population to the discovery study. The incomplete coverage of independent loci that were discovered due to lack of adequate representation on the target study genome-wide microarray, however, is a specific cause that can be empirically characterized.

In this project, we evaluate the presence or absence of the 88 previously identified variants that were carried forward for replication in Grove et al. on standard microarrays before quality control (QC), after QC and post-imputation. To accomplish this, we use genome-wide data from three studies of familial ASD and one population-based case–control study. Collectively, these studies used a diversity of microarrays spanning approximately one million to more than five million sites. We then expand our evaluation of coverage in each study to a genome-wide PGS approach using a novel method and report on our findings.

## Methods

### Familial ASD studies

Individuals included in our analyses were participants in one of three studies of high familial likelihood for ASD. These studies were designed to investigate the early life factors involved in ASD and neurodevelopmental outcomes by enrolling mothers who had already given birth to a previous child with a clinical ASD diagnosis. The Early Autism Risk Longitudinal Investigation (EARLI) Study [21] and Markers of Autism Risk in Babies-Learning Early Signs (MARBLES) [22] both enrolled mothers who already had a child with ASD to participate when they became pregnant with another child and followed them throughout pregnancy until 36 months of age. We also included participants from the Infant Brain Imaging Study (IBIS), [23–25] which enrolled these high likelihood mothers and infants at 6 months and were then re-evaluated at 12 and 24 months of age.

### Population-based study to explore early development phase I

A total of 3,769 children were enrolled in the Study to Explore Early Development, Phase 1 (SEED 1), a multisite cohort initiative designed to obtain a representative sample of ASD and typically developing preschool-aged children in the US. Children between 2 and 5 years old, born between September 1, 2003, and August 31, 2005, and living in one of six study site vicinities (San Francisco Bay Area, Philadelphia metropolitan area, northeast Maryland, central North Carolina, and the Atlanta metropolitan area) were ascertained through a variety of methods, including diagnostic clinics, organizations providing evaluation or services for children with developmental problems, educational departments, and population vital records. Detailed recruitment procedures are described elsewhere [26].

### Genetic data

#### Cleaning and imputation

Samples from familial ASD cohorts were genotyped using the MEGA 1-Million (M)(MARBLES) and 5 M Illumina (EARLI and IBIS) chips. The Multi-Ethnic Global Array (MEGA) is a high-density array consisting of more than 1.7 million single nucleotide polymorphisms (SNPs) and is designed to represent diverse ancestries. The 5 M array is more comprehensive, consisting of around 5-million high-density SNPs, and has high overlap with the 1 M array. The SEED samples were genotyped on the more recently available Global Screening Array (GSA), which contains 640 K variants and represents diverse ancestry. For all studies, whole blood or buccal tissue was collected and samples were processed and stored. Genotyping was performed at the Johns Hopkins Genetic Resources Core Facility (GRCF).

The resulting genotypes were then subject to quality control filters according to standard criteria [27]. Briefly, using PLINK v1.9 [28], individuals failing QC checks for sex, relatedness, sample call rate of 0.03 (using –missing), and divergent ancestry were removed. SNPs were also removed based on the following criteria: MAF ≥ 0.05 among European samples, missing call rates exceeding 0.05, and Hardy–Weinberg equilibrium exact test *p*-value below 0.00001 among European samples.

Following cleaning, studies were imputed on the Michigan Imputation Server using the Minimac4 pipeline provided by the University of Michigan. We specified the 1000 genomes project (1000G) Phase 3 v5 reference panel, hg19 array build, Eagle v2.4 phasing [29], and the quality control and imputation mode. We imputed each target study separately and included 2504 1000G samples along with the target samples surviving QC. Post-imputation, we required imputed SNPs to correlate with the true unobserved genotypes at an r-squared (R^2^) > 0.80.

#### Genetic ancestry classification

After applying both the variant and sample level filters, measured genotypes were used to compute genetic ancestry variables from principal components analysis in Eigensoft [30] according to a recommended procedure [27], which includes pooling the target samples with the 2504 1000G samples from diverse ancestral groups [31].

Classification to a defined ancestral group was carried out using K-means (R v4.0.3 with “kmeans” function) based on the first 2 principal components (PC1 and PC2) resultant from this procedure. Only the 661 African, 504 East Asian, and 503 European samples from 1000G were used as anchors to define three ancestral clusters. The minimum, maximum and standard deviation of PC1 and PC2 for each of the three 1000G ancestry groups were computed. Target sample principal components were then compared to these values to classify into an ancestral group. European ancestry classification required that the target PC1 and PC2 value fell within 1.96 standard deviations of the minimum and maximum values for the 1000G European corresponding principal components. All other ancestries were classified as non-European.

#### Top ranking ASD discovery SNPs

This analysis uses the top 88 discovery variants by *p*-value that were implicated in the ASD discovery GWAS [12]. The discovery GWAS combined samples from the Danish iPSYCH population as well as samples from the Psychiatric Genomics Consortium (PGC), totaling 18,381 cases of ASD and 27,969 controls reflecting European ancestry. Following initial identification of 88 top loci, a replication analysis was conducted with another 2,119 cases of ASD and 142,379 controls pooled across five different populations of Northern European ancestry. The three identified loci were found to be significant, while two additional loci became significant when meta-analyzed with the discovery sample. Although the majority of the single variant tests did not achieve statistical significance, a test to replicate the direction of effects was significant. For each identified top variant, the minor allele frequency, *p*-value, and odds ratios were provided in Supplementary Table 1.

#### Identifying correlated SNPs

In addition to the original list of 88 top variants, nearby SNPs that were highly correlated with the discovery index variants were identified as proxies. We accomplished this by accessing the GRCH37 REST API database (http://grch37.rest.ensembl.org) via R software. The Ensemble database uses the 1000G phase 3 reference to perform searches for correlated SNPs in specific windows. Proxy searches were performed specifying a reference panel made up of samples with European ancestry. Proxies were kept if they were found to be in linkage disequilibrium (LD) with the index SNP at R^2^ >  = 0.80. When multiple proxies were available for a missing SNP, the proxy was selected based on highest R^2^ with the index SNP and closest physical distance.

### Calculating single variant coverage among top ASD hits

Representation of the original index or proxy SNP was determined for cleaned and imputed target datasets by evaluating the overlap using chromosome, base pair, and variant identifier (rs number). An identical process was applied to obtain coverage for the Global Diversity Array-8 (GDA) and the Infinium PsychArray. Because we did not have target samples typed on these arrays, we were only able to evaluate coverage for variants on the pre-cleaned manifest files. The manifest files we downloaded for evaluation are publicly available on the Illumina website ( [1, 2]).

### Literature search for published reports using an ASD polygenic score

To characterize the methods for deriving and describing ASD-PGS that are commonly employed by researchers to date, we conducted a literature search in PubMed to identify manuscripts published through October 2022 that reported on ASD-PGS associations, where the target sample was in children and the outcome was either ASD or an ASD-related trait. We searched on the terms “ASD” and “Polygenic Risk Score” to obtain 109 potential hits. We also supplemented our search with the same terms in Google, evaluated the suggested literature from each identified manuscript, and considered references returned by the PGS Catalog [32] when searching for “autism”. Two researchers evaluated each abstract to rule out those studies that did not report on an ASD-PGS, did not perform analyses in children, or did not report on child ASD status or child ASD trait. We did not consider randomized trials. After a review of abstracts, 36 potential manuscripts met our criteria, and of these, 24 were selected for extraction. Extraction included the method and software used to derive ASD-PGS, parameters specified for the method, and whether the number of SNPs informing the ASD-PGS was reported.

### Creation of an information metric for polygenic scores

While there exist several methods to derive PGS, the majority of researchers employ the clumping and thresholding (C + T) method [33], where redundant SNPs due to linkage disequilibrium (LD) are removed (i.e. clumping), and only the SNPs that fall below an established discovery *p*-value threshold level are included in the score (i.e. thresholding). Scores are derived using PLINK software [28, 34], which can also be implemented via PRSice version 1 [35] or version 2 [36]. To derive a score, the researcher must specify: 1) a reference panel, 2) a target panel and 3) a target population. The reference panel will be used to determine the amount of LD between nearby SNPs, supervised by discovery effect sizes or *p*-values, to make decisions about which SNPs to prune in the “clump” procedure. The target panel will limit the choice of SNPs to those available after cleaning and/or imputing the target array data. Thus, together, the reference and target panels will incorporate the discovery information to select the list of SNPs that inform the PGS. Finally, using the clumped SNP list, the “score” command will sum and weight each genotype for each sample in the target population.

With this C + T method, if a SNP, or any proxy SNP, is not available to represent a genomic region, its failure to be incorporated into the PGS calculation is likely to result in information loss. Even in the case when a proxy SNP is selected from the target panel to represent the index discovery SNP, some information loss will occur. Our goal was to assess the impact of these missing representations of discovery loci due to lack of coverage when using a C + T method to derive ASD-PGS, even after high quality imputation recovered a number of SNPs in the target data that were not present on the cleaned, measured GWAS array.

**Fig. 1:**
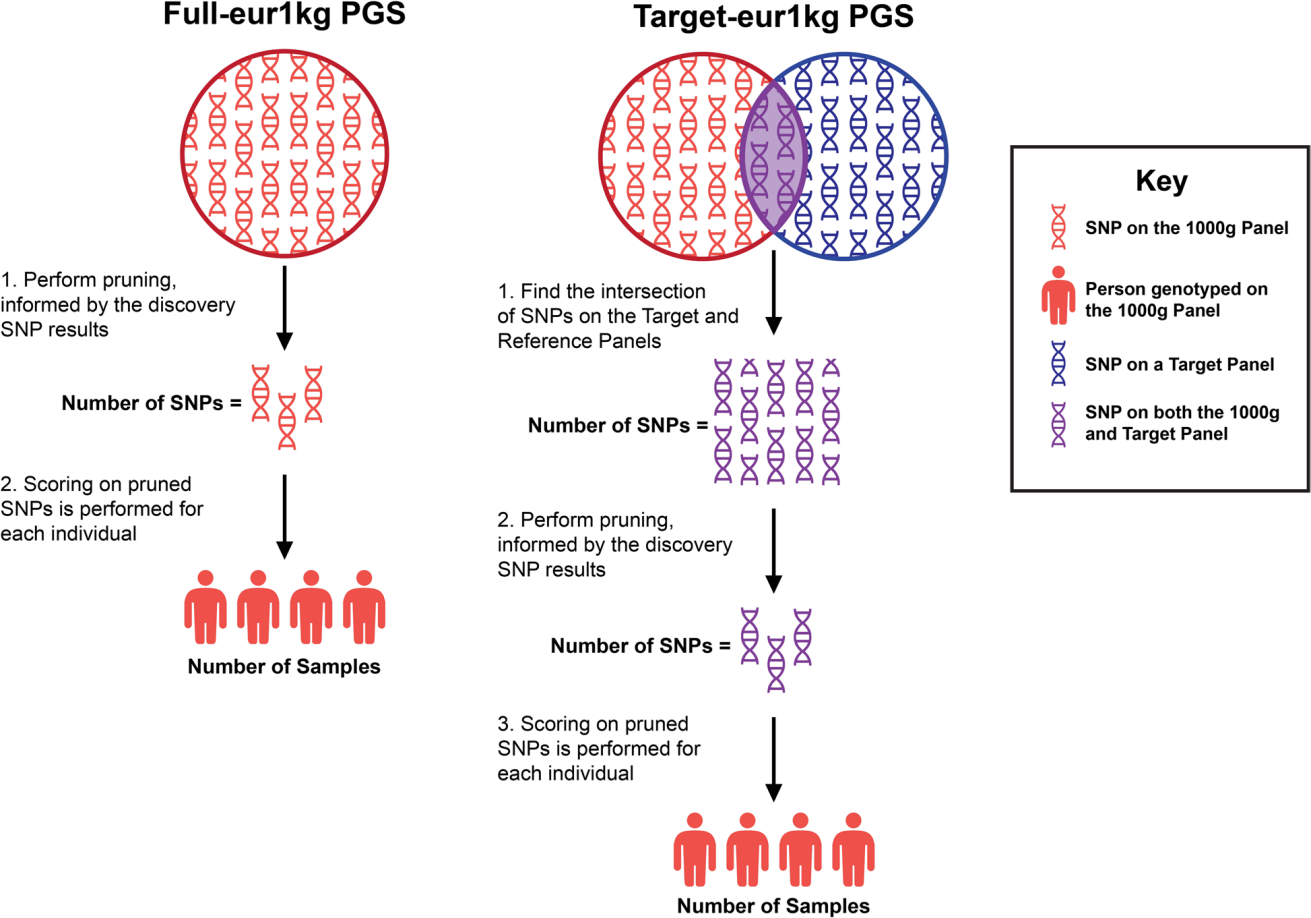
PGS Computation Workflow. Legend: Our genome-wide information metric is simply the direct correlation between full-eur1kg and target-eur1kg PGS, which reflects information loss genome-wide. A correlation of 1.0 indicates that no information loss occurred, whereas a low correlation suggests a substantial loss of information. We derive each ASD-PGS using a C + T method, limiting to biallelic, high quality (info > 0.80) SNPs, for a suite of *p*-value discovery thresholds (5 × 10^–8^, 1 × 10^–6^, 10^–4^,10^–3^, .01, 0.05, 0.10, 0.20, 0.50 and 1.0) scored in PLINK software. Because target-eur1kg-ASD-PGS were clumped and scored for each cohort separately, a different SNP selection informed each of the target-eur1kg PGS, leading to cohort-specific correlations with the full-eur1kg PGS

To accomplish this, we made use of the 1000G phase 3 v5 reference panel [31], which contains a comprehensive set of genetic variants from whole genome sequencing across 26 different populations. We limited the reference panel to the 498 unrelated individuals of European ancestry (eur1kg). The eur1kg panel can serve as the reference panel when clumping, and can also act as a high coverage, comprehensive target panel, in addition to serving as the target population. Alternatively, the high quality post-imputed data from a target cohort can also serve as a target panel, and the samples can be used as the target population in whom scoring takes place. We compute two different PGS as outlined in Fig. 1. All PGS are informed by the Grove et al. discovery results, and the eur1kg panel is always specified as the reference panel of choice. The first score represents the score computed from a comprehensive panel and specifies the eur1kg as both the target panel and the target population (full-eur1kg PGS); the process to create full-eur1kg PGS is shown in Fig. 1 on the left. The second score represents the PGS computed using the cohort target panel, which is not as comprehensive as eur1kg. This second PGS is scored in the eur1kg sample to serve as an “apples to apples” comparison to the full-eur1kg PGS, as shown on the right of Fig. 1 (referred to as target-eur1kg PGS). Our coverage metric is the correlation between the full-eur1kg and target-eur1kg PGS, which will range from 0.0 to 1.0.

## Results

### Characteristics of the target cohort samples

The total number of samples, as well as sex and ancestry distributions per target cohort, are provided in Table 1. The samples include ASD children, parents and siblings. A large majority of the samples reflect European ancestry in both the IBIS (88.5%) and SEED (69.6%) studies, while EARLI (56.4%) and MARBLES (54.5%) are slight majority European. All cohorts had < 10% of the sample East Asian and African, but admixed individuals make up a sizeable minority (ranging from about 10% to 30%). EARLI and IBIS have a more balanced sex proportion than MARBLES and SEED, which have predominantly female samples. The total number of samples across all cohorts ranges from 633 to 914 individuals.

**Table 1:** Characteristics of target study participants

Characteristic	Type	EARLI	IBIS	MARBLES	SEED
**N**	-	827	914	633	863
**Sex (%)**	Male	461 (55.7)	529 (57.9)	177 (27.9)	295 (34.1)
	Female	366 (44.2)	385 (42.1)	456 (72.0)	568 (65.8)
	European	467 (56.5)	809 (88.5)	345 (54.5)	601 (69.6)
	Non-European	360 (43.5)	105 (11.5)	288 (45.5)	262 (30.4)

### Characteristics of top 88 variants

Grove et al. (2019) provided metrics for the 88 variants selected for follow up from their discovery efforts, and we provided the ranges reported on allele frequency and effect sizes in Supplementary Table 1. All minor allele frequencies for each of the top 88 variants were > 1%. The odds ratios for the variants reflected moderate to modest effect sizes, ranging from 0.658 to 1.342. Of the total number of SNPs, 70 (79.55%) were bi-allelic. As expected, all 88 variants exhibited suggestive statistical significance (*p*-values < 1 × 10^–5^) in the original analysis, with 53 of the 88 variants also achieving significance in the follow-up study [12].

### Coverage of top ASD discovery variants

The surviving number of SNPs and coverage among the 88 variants identified via discovery are provided in Table 2 for clean, measured genotypes and for high quality (R^2^ > 0.80) post-imputation genotypes. Considering the clean, measured genotypes, no study panel we examined directly covered all 88 of the top variants. For the IBIS and EARLI studies, which were both genotyped using the 5 M arrays, 31 (35%) and 32 (36%) variants were represented, respectively. MARBLES (1 M array) and SEED (GSA array) only contained 11 of the index variants. Including proxy SNPs improved coverage for measured genotypes across all studies, with 52 of the index discovery variants represented in IBIS, 54 represented in EARLI, 28 in MARBLES and 31 in SEED. Once we considered high quality imputed variants, coverage for IBIS and EARLI cohorts reached 100%, but MARBLES was still missing 6 of the 88 variants and SEED 5 of the 88 variants.

**Table 2:** Sample size, variant numbers, and coverage statistics for top ASD hits per cohort

**Cohort** (Array)	**N measured Variants surviving QC**	**N Variants surviving post-imputation QC**	**N Top ASD Variants on GWA Array**^a^	**N Top ASD Variants surviving post-imputation QC**	**N SNPs in Score**^b^	**ASD-PGS coverage metric**^c^
**IBIS** (5 M)	2,400,509	38,343,801	52 (31)/88	88/88	249,698	0.9569
**EARLI** (5 M)	2,525,262	38,123,095	54 (32)/88	88/88	242,199	0.9445
**MARBLES (1 M)**	578,578	33,317,727	28 (11)/88	82/88	229,119	0.9295
**SEED** (GSA)	877,115	32,275,019	31 (11)/88	83/88	268,035	0.9849

^a^Coverage reflects the presence on the panel of either the discovery variants itself or at least one proxy SNP. Number in parentheses refers to the non-proxy number

^b^Full-eur1kg containes 281,593 SNPs after clumping

^c^Coverage metric reflects the correlation of full-eur1kg and target-eur1kg scores as explained in Fig. 1

We also analyzed coverage of the top 88 variants and their proxies on the GDA and iPSYCH arrays using publicly available manifest files. Initial analysis of the GDA array revealed representation of 16 (18%) of the top 88 variants, and improved to 38 (43%) when including proxy variants (see Supplementary Table 2 for full proxy list). Analysis of iPSYCH yielded 11 of the original 88 discovery variants on the array, and the inclusion of proxy SNPs improved coverage to 25 (28% of all 88 variants).

### Findings from a literature search for ASD-PGS associations

Our literature search identified 24 published manuscripts with reports of ASD-PGS with ASD or ASD-related traits in children, and extracted information from these manuscripts is in Supplementary Table 3. Almost all microarrays were from Illumina and ranged from the 550 Quad chip to the more recent GSA chips. In all verifiable studies, PGS were made from imputed genotypes. Four studies employed a method other than C + T to derive ASD-PGS. Among those using C + T, we observed a range of specified r^2^ for LD correlation and window sizing, and about half employed a version of PRSice to carry out scoring. Only one study employed both long- and short-range pruning. Importantly, among the 24 studies, 16 reported the number of SNPs that informed the ASD-PGS, however, no alternate metric to reflect information loss due to SNPs that weren’t directly represented or indirectly represented by proxy in the score was reported.

### Genomic coverage using ASD-PGS

**Fig. 2:**
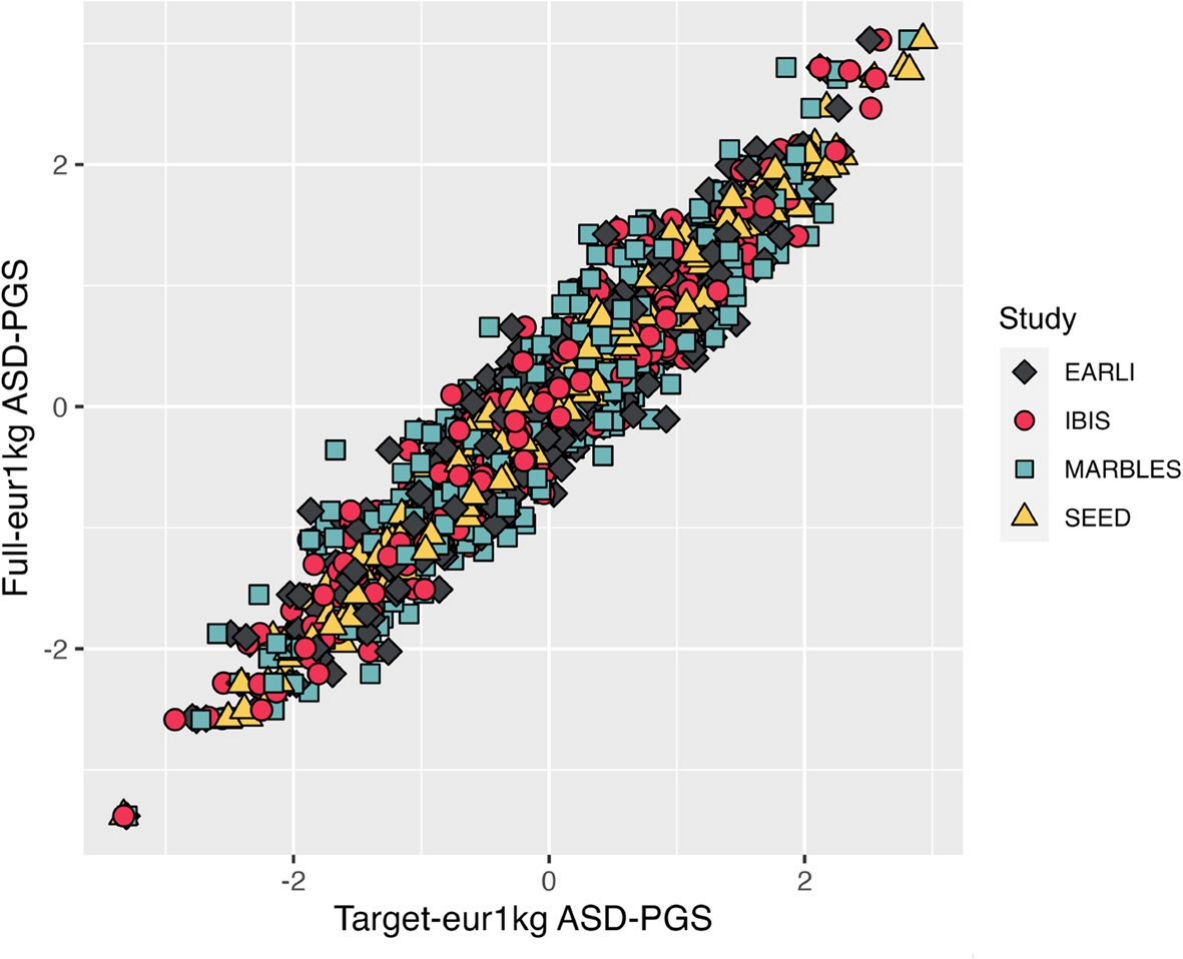
Correlation between Full-eur1kg ASD-PGS and Target-eur1kg ASD-PGS for each of the 498 eur1kg samples. Legend: A separate Target-eur1kg ASD-PGS was made in each of the four target cohorts, reflecting the different SNP selection that emerged from the pruning process. “The correlation coefficient for each study using this data reflect the ASD-PGS coverage metric reported in the last column of Table 2.” The Full-eur1kg is the same for each cohort. All scores represent a discovery *p*-value threshold of 1.0

To determine potential loss of information in the ASD-PGS from unrepresented or indirectly represented variants across the genome, we correlated two different PGS, both derived in the 1000G sample, using complete SNP data and again using only the available SNP target data, as explained in the Methods and depicted in Fig. 1. Figure 2 shows a scatter plot between the eur1kg-full-PGS and the eur1kg-target-PGS, color coded for each high ASD likelihood target cohort and the SEED 1 target cohort. The two different PGS informing our metric exhibited a linear relationship with each other for all target cohorts, confirming that a correlation is an appropriate comparison statistic. In general, correlation was high for all study platforms, ranging from 0.9295 to 0.9849, using the most liberal discovery *p*-value threshold of 1.0 (see last column Table 2).

**Fig. 3:**
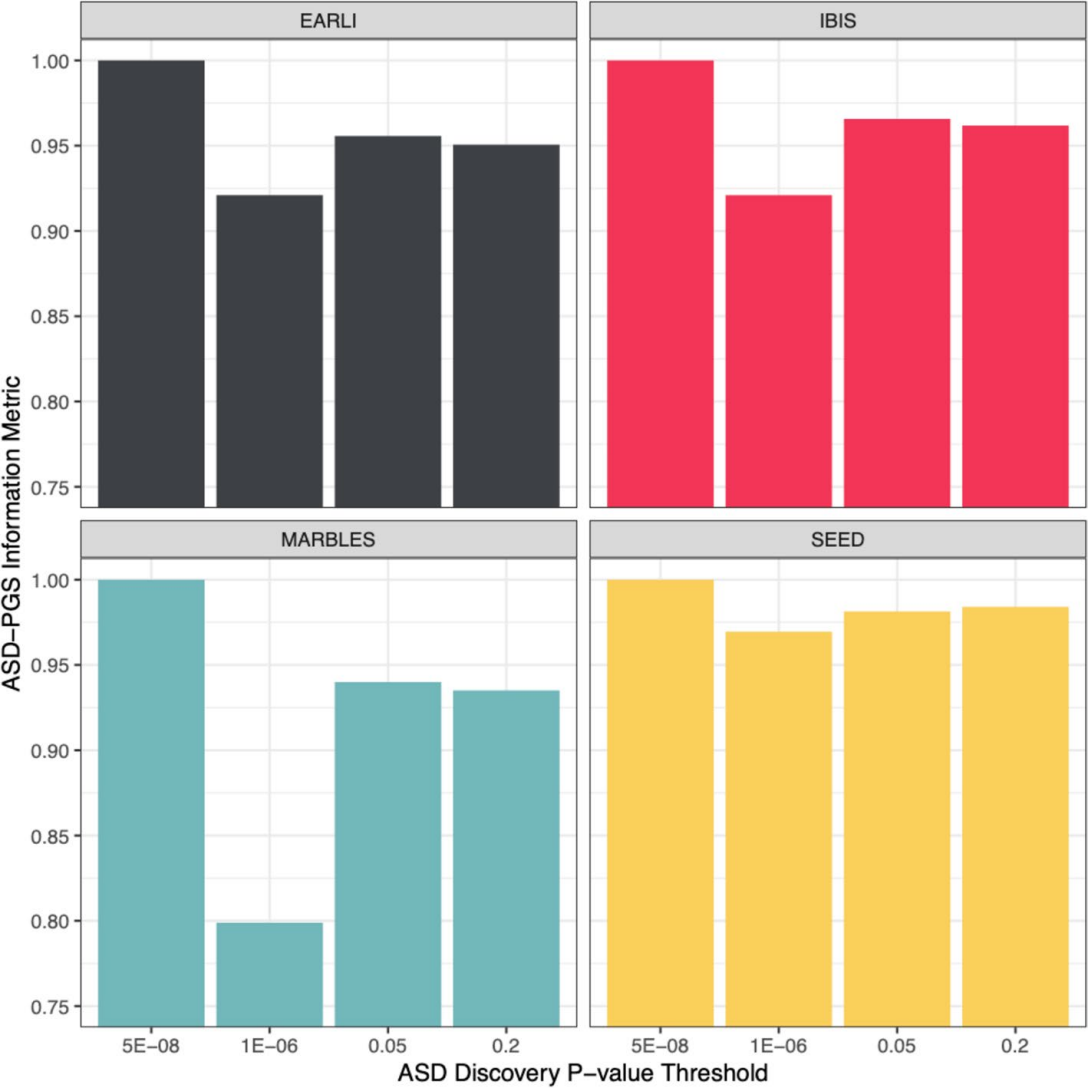
The correlation metric (Full-eur1kg ASD-PGS versus Target-eur1kg ASD-PGS) representing loss of information in each of the four target cohorts. Legend: The metric represents information loss or genome-wide coverage from using a pruning and thresholding derivation method in each of the four target cohorts. Thresholds for discovery *p*-values are genome-wide significant (5 × 10^–8^), genome-wide suggestion (1 × 10^–6^), 0.05 and 0.20

Figure 3 shows correlations between the full and target PGS by select discovery *p*-value thresholds. We consistently saw a drop in our PGS coverage metric for the suggestive discovery *p*-value threshold of 1 × 10^–6^, but coverage improved thereafter, suggesting that coverage is particularly low for ASD in the statistically suggestive range. Results for all *p*-value threshold levels are provided in Supplementary Table 4. We did not observe clear patterns between PGS coverage and platform array density or for the number of SNPs informing the ASD-PGS.

## Discussion

We evaluated coverage of top GWAS hits for ASD for a number of Illumina microarrays. We used available genotype data from the Illumina 5 M, MEGA and GSA arrays, plus two arrays using publicly available manifest files. The modern GWA arrays increasingly used in genetic research include the Illumina PsychArray (iPSYCH), Global Diversity Array (GDA) and Global Screening Array (GSA). These modern arrays are less costly compared to the denser (5 M or 2.5 M) arrays. They are also designed to expand on the intent of the Multi-ethnic arrays (MEGA or 1 M), which included variants meant to tag diverse ancestries and improve imputation performance. The modern arrays continue to represent diverse ancestry while providing content for an increasing number of clinical traits or disease outcomes. Our evaluation of coverage determined that many variants are not directly represented. For GSA, 5 M and MEGA chips, less than half of the 88 variants identified in large-scale GWA for ASD were directly represented. Using publicly available manifest files, we observed similar low coverage for the iPSYCH and GDA arrays as well. Further, when considering proxy SNPs that are highly correlated with the index variant, we still observe incomplete coverage for all arrays.

We then re-calculated coverage using available study genotype data after standard QC procedures for GSA, MEGA and 5 M arrays, and confirmed that coverage for measured genotyping is incomplete. Perhaps most importantly, we identified that for the dense 5 M array, high quality imputed data can bring the coverage to 100%, but for less dense arrays, while providing high coverage, high quality imputation can still fall short of completion. These findings suggest that attempts to recover lost SNPs are not guaranteed to be successful, and that different microarrays can yield varying levels of coverage.

The results of our literature search provide a gestalt for the state of current research practices when deriving ASD-PGS in target study data. We found that the vast majority of studies used a C + T method rather than using alternative methods, such as LDPred [37], that may somewhat improve variance explained [38]. Papers published more recently, however, are beginning to employ these newer methods. The C + T approach, however, is still likely to continue to be a popular approach due to its intuitive appeal, lower computational requirements, and the fact that it can be scored in PLINK software, which is familiar to many researchers. We also observed that though software and parameter specification for C + T varied somewhat, the general procedures and software were quite similar across studies. Importantly, the only metric reported across these studies that reveal insight into the coverage of the target study ASD-PGS was the total number of SNPs incorporated into the score, highlighting the gap in current research practice for evaluating information loss. It is also notable that one-third of the studies did not report any metric yielding some insight into SNP coverage for the ASD-PGS. Interestingly, the University of Michigan Imputation Server is offering a beta version to derive PGS for hundreds of different traits, using discovery findings published in the PGS Catalog [32], but appears to only offer the total number of index SNPs contained in the score as a measure of PGS coverage.

We then explored a simple and novel approach that would intuitively provide an indication of information loss. Our metric, in addition to addressing the selection of SNPs, reflects the extent to which this imperfect or missing information is influenced by observed allele frequencies and weighted by the discovery effect size, so that the joint impact of all these factors on the PGS is incorporated into the measure. Thus, we provide an intuitive, scalar measure that reflects the true loss of information genome-wide from multiple sources that is not readily intuitive by reporting the total number of SNPs.

To compute our PGS coverage metric for ASD, we use the eur1kg reference panel. For the ASD trait, our choice to compute the full-eur1kg and target-eur1kg scores in a European panel is appropriate because reference and discovery ancestry should reflect each other [39], and the ASD discovery is comprised almost entirely of individuals of European descent. There exist many alternative reference panels that offer a comprehensive set of variants via sequencing to calculate a PGS coverage metric. For example, the Haplotype Reference Consortium (HRC) [40] may provide better imputation accuracy, particularly for rare variants, due to its larger sample size compared to the 1000G European populations [41]. Several panels are also available to researchers representing African ancestry including the Consortium on Asthma among African ancestry Populations in the Americas (CAAPA) [42]**,** the African Genome Resources (AGR) (https://www.apcdr.org/) and African Genome Variation Project (AGVP) [43]. The selection of reference panel to compute a coverage metric will depend on the ancestry of the discovery population as well as consideration for the ancestry of the target population.

Our PGS metric can serve as a useful tool for researchers. Current published studies for ASD-PGS focus on the association with ASD outcomes for the purpose of gaining insight into the disorder’s genetic etiology. With the establishment of the PGS Catalog and the opportunity to compute PGS via the Michigan Imputation Server based on the PGS Catalog discovery input, focus may shift to creating genetic scores in target samples that are derived from pre-prepared, filtered and pruned SNP lists from a standard reference population. In this case, the target sample PGS value can be placed directly along the distribution of PGS in the reference population to determine if a target individual has a high, average, or low genetic load, rather than relying on the relative ordering of samples within a target study. If index SNPs are not directly genotyped and/or not surviving pre-imputation QC procedures, then knowledge about how this potentially influences coverage would be important to calculate and report. As PGS methods and discovery findings develop for ASD and other traits, we argue that the reporting of an intuitive coverage metric that captures lost discovery information should become an essential part of any future PGS effort.

### Limitations

There were several limitations to our study. First, although we present coverage prior to cleaning and imputation, using Illumina manifest files for several arrays, we did not measure our own genotypes using either of the GDA or iPSYCH arrays. A lack of in-hand data for these studies prevented us from assessing top hits coverage after cleaning and imputation procedures were applied as well as from computing our PGS coverage metric. These arrays, however, have high overlap with the GSA, so our findings using the SEED data, which was genotyped on the GSA, may provide a good estimate of the GDA and iPSYCH coverage. Second, our analysis is in studies with modest sample sizes, and additional evaluation with studies ranging from a small to large number of samples may offer the opportunity to better explore trends in coverage. Third, our computation of top variant coverage as well as PGS coverage could have been influenced by the presence in our target cohort samples of non-Europeans via any influence these samples may have had on the QC and imputation process. To acknowledge this influence, we computed minor allele frequency and Hardy Weinberg Equilibrium *p*-values in separate ancestries before applying filtering criteria. When imputing, we included all 1000G ancestries along with our target samples and impute to the full multi-ethnic 1000G panel. Despite differing LD pattern between ancestries, previous research has suggested that this strategy of imputing to a diverse ancestry panel can result in higher imputation accuracy [44, 45], and thus, the presence of non-European target samples should have little to no influence on our coverage metric. Fourth, the PGS coverage metric was computed in Europeans, and is not generalizable to non-European ancestries. Although beyond the scope we have defined here, large-scale GWAS in non-European ancestries are available for some traits and can be used to explore PGS coverage. Finally, we limit our approach to the clumping and thresholding method of PGS derivation, but extensions to LDpred2 [37, 39] and other derivation methods including SBayesR [46], SDPR [47], PRS-CS [48] and others may be possible.

## Conclusions

In summary, we provide insight into the coverage of top ASD GWAS variants for a number of commonly used genome-wide microarrays. We also create and apply a genome-wide coverage metric to assess how well the ASD-PGS in a particular target sample is incorporating the available information from the discovery GWA results. While applied only to ASD here, our approach can be used for any trait with available discovery results and offers a more intuitive and satisfying alternative to reporting the total number of SNPs included in the PGS. There may be natural extensions of our metric for other PGS derivation methods beyond the clumping and thresholding approach that can be explored in future research.

## Supplementary Information

Additional file 1: Supplementary Table 1. Characteristics of ASD Discovery GWA Top 88 Variants. Contains information about the MAF, odds ratio, p values, and number of bi-allelic SNPs in the original 88 variants identified by Grove et al.

Additional file 2: Supplementary Table 2. List of Proxy SNPs to the Top 88 Variants from ASD Discovery GWAS. Contains the list of proxy SNPs for the top 88 variants ordered by chromosome with corresponding rs ID, R^2^, average MAF, and distance in base pairs.

Additional file 3: Supplementary Table 3. Characteristics of ASD-PGS Derivation in Reported Studies. Contains the results of the literature search on characteristics of ASD-PGS derivation in 24 studies, including information from the following categories: ASD Discovery GWAS, Target GWA Chip, Imputed/Reference Panel, Post-imputation filters, PRS software, Clumping reference panel, Clump r^2^, clump window size, presence of a 2nd clump round, specification of a PRS threshold, and whether # of SNPs was reported.

Additional file 4: Supplementary Table 4. PGS coverage metric by Discovery *P*-value threshold. Contains values of our novel PGS coverage metric for IBIS, EARLI, MARBLES, and SEED for 9 selected *P*-values.

## Data Availability

The SEED 1 data in this study are not publicly available due to lack of explicit consent for such sharing in the written informed consents for SEED sites, per the CDC IRB that governs the SEED network. Discovery GWAS results are available in the study by Grove et. Al. The datasets generated during the EARLI, IBIS, and MARBLES studies are not publicly available but are available from the corresponding author on reasonable request. 1000 Genome Data is publicly available.

## Abbreviations

ASD: Autism Spectrum Disorder
PGC: Psychiatric Genomics Consortium
MAF: Minor Allele Frequency
PGS: Polygenic Score
ASD-PGS: Polygenic Score for Autism Spectrum Disorder
SNP: Single Nucleotide Polymorphism
1000G: 1000 Genomes
EARLI: Early Autism Risk Longitudinal Investigation
IBIS: Infant Brain Imaging Study
MARBLES: Markers of Autism Risk – Learning Early Signs
SEED: Study to Explore Early Development
HRC: Haplotype Reference Consortium
GDA: Global Diversity Array
GSA: Global Screening Array
iPSYCH: Infinium PsychArray
QC: Quality Control
MEGA: Multi-Ethnic Global Array
