# Supplementary material for: Commonly used genomic arrays may lose information due to imperfect coverage of discovered variants for autism spectrum disorder
Source: J Neurodev Disord. 2024 Sep 12;16:54. doi: 10.1186/s11689-024-09571-8 (PMC11397030; doi:10.1186/s11689-024-09571-8)
Supplement: Supplementary file 1 — Additional file 1: Supplementary Table 1. Characteristics of ASD Discovery GWA Top 88 Variants. Contains information about the MAF, odds ratio, p values, and number of bi-allelic SNPs in the original 88 variants identified by Grove et al. [file 11689_2024_9571_MOESM1_ESM.docx]

| **Supplementary Table 1. Characteristics of ASD Discovery GWA Top 88 Variants** | | |
| --- | --- | --- |
|  | **Min** | **Max** |
| **MAF (Cases)** | 0.019 | 0.987 |
| **MAF (Controls)** | 0.016 | 0.989 |
| **Odds Ratio** | 0.658 | 1.342 |
| **Odds Ratio –follow up study** | 0.692 | 1.524 |
| **Meta Odds ati** | 0.701 | 1.332 |
| **P** | 1 x 10^-5^ | 2 x 10^-9^ |
| **% non-bi-allelic SNPs** | 20.45% | |
