# Supplementary material for: Commonly used genomic arrays may lose information due to imperfect coverage of discovered variants for autism spectrum disorder
Source: J Neurodev Disord. 2024 Sep 12;16:54. doi: 10.1186/s11689-024-09571-8 (PMC11397030; doi:10.1186/s11689-024-09571-8)
Supplement: Supplementary file 2 — Additional file 2: Supplementary Table 2. List of Proxy SNPs to the Top 88 Variants from ASD Discovery GWAS. Contains the list of proxy SNPs for the top 88 variants ordered by chromosome with corresponding rs ID, R2, average MAF, and distance in base pairs. [file 11689_2024_9571_MOESM2_ESM.docx]

Supplementary Table 2. List of Proxy SNPs to the Top 88 Variants from ASD Discovery GWAS

* Average SNP MAF for EARLI, IBIS, and MARBLES studies

| Rank Order | SNP | Proxy SNP | R^2^ | AvgMAF* | Distance(bp) |
| --- | --- | --- | --- | --- | --- |
| 1 | rs910805 | rs809220 | 0.843099 | 0.20129 | 165921 |
| 2 | rs10099100 | rs7820334 | 0.842916 | 0.25276667 | 3073 |
| 3 | rs71190156 | No Proxy |  |  |  |
| 4 | rs6047270 | rs4815021 | 0.886905 | 0.42301667 | 1669 |
| 4 | rs6047270 | rs3762194 | 0.868629 | 0.39625667 | 4511 |
| 4 | rs6047270 | rs6047279 | 0.864079 | 0.42018667 | 20141 |
| 4 | rs6047270 | rs6047293 | 0.864079 | 0.42055 | 32275 |
| 4 | rs6047270 | rs2145100 | 0.864079 | 0.423 | 41587 |
| 4 | rs6047270 | rs6035795 | 0.85956 | 0.36720667 | 23673 |
| 4 | rs6047270 | rs6047274 | 0.85956 | 0.37652333 | 12914 |
| 4 | rs6047270 | rs6047273 | 0.855482 | 0.39365667 | 12587 |
| 4 | rs6047270 | rs6047280 | 0.850194 | 0.40619 | 21723 |
| 4 | rs6047270 | rs2236178 | 0.841597 | 0.33237333 | 20601 |
| 4 | rs6047270 | rs6113163 | 0.822994 | 0.41304333 | 65748 |
| 4 | rs6047270 | rs754332 | 0.822994 | 0.42323667 | 55686 |
| 4 | rs6047270 | rs6137292 | 0.815198 | 0.41851667 | 73591 |
| 4 | rs6047270 | rs6137286 | 0.809434 | 0.35955 | 57540 |
| 4 | rs6047270 | rs1569959 | 0.80099 | 0.37859 | 101198 |
| 4 | rs6047270 | rs6137297 | 0.80099 | 0.39738333 | 95811 |
| 4 | rs6047270 | rs6047320 | 0.80099 | 0.39979 | 96737 |
| 4 | rs6047270 | rs6035817 | 0.80099 | 0.40124667 | 99092 |
| 4 | rs6047270 | rs6082351 | 0.80099 | 0.41319 | 91632 |
| 4 | rs6047270 | rs2424353 | 0.80099 | 0.42044333 | 99833 |
| 5 | rs111931861 | No Proxy |  |  |  |
| 6 | rs2391769 | No Proxy |  |  |  |
| 7 | rs138867053 | No Proxy |  |  |  |
| 8 | rs183563276 | No Proxy |  |  |  |
| 9 | rs1452075 | rs7429220 | 0.845173 | 0.15678667 | 1825 |
| 9 | rs1452075 | rs57384528 | 0.831101 | 0.20269 | 11109 |
| 10 | rs1222063 | No Proxy |  |  |  |
| 11 | rs142920272 | rs8070723 | 0.889376 | 0.13745 | 220776 |
| 11 | rs142920272 | rs4383188 | 0.889376 | 0.11731667 | 104237 |
| 11 | rs142920272 | rs56301633 | 0.889376 | 0.11207333 | 223990 |
| 11 | rs142920272 | rs1078269 | 0.889376 | 0.11207333 | 226003 |
| 11 | rs142920272 | rs17652121 | 0.889376 | 0.11207 | 227867 |
| 11 | rs142920272 | rs62063845 | 0.889376 | 0.11207 | 230546 |
| 11 | rs142920272 | rs62063786 | 0.889376 | 0.11207 | 240817 |
| 11 | rs142920272 | rs62063857 | 0.889376 | 0.11206667 | 225175 |
| 11 | rs142920272 | rs10445337 | 0.889376 | 0.11206333 | 234440 |
| 11 | rs142920272 | rs62063787 | 0.889376 | 0.11206333 | 240804 |
| 11 | rs142920272 | rs1981997 | 0.889376 | 0.11177 | 245073 |
| 11 | rs142920272 | rs1800547 | 0.889376 | 0.11176667 | 249994 |
| 11 | rs142920272 | rs17577094 | 0.889376 | 0.11095667 | 114348 |
| 11 | rs142920272 | rs370558872 | 0.889376 | 0.11094667 | 215114 |
| 11 | rs142920272 | rs1117253 | 0.889376 | 0.11093333 | 152543 |
| 11 | rs142920272 | rs12150090 | 0.889376 | 0.11092 | 185954 |
| 11 | rs142920272 | rs1468241 | 0.889376 | 0.11091333 | 105687 |
| 11 | rs142920272 | rs75534191 | 0.889376 | 0.11091333 | 210116 |
| 11 | rs142920272 | rs12150447 | 0.889376 | 0.11090667 | 173715 |
| 11 | rs142920272 | rs34579536 | 0.889376 | 0.11090667 | 192934 |
| 11 | rs142920272 | rs17574228 | 0.889376 | 0.11090667 | 197331 |
| 11 | rs142920272 | rs2158257 | 0.889376 | 0.11090667 | 197497 |
| 11 | rs142920272 | rs16940806 | 0.889376 | 0.11090667 | 198014 |
| 11 | rs142920272 | rs17574040 | 0.889376 | 0.11090667 | 198975 |
| 11 | rs142920272 | rs1052587 | 0.889376 | 0.11090667 | 199236 |
| 11 | rs142920272 | rs17574604 | 0.889376 | 0.11086 | 190227 |
| 11 | rs142920272 | rs17652961 | 0.889376 | 0.11086 | 193485 |
| 11 | rs142920272 | rs17574361 | 0.889376 | 0.11086 | 193638 |
| 11 | rs142920272 | rs78681971 | 0.889376 | 0.11086 | 195621 |
| 11 | rs142920272 | rs80028338 | 0.889376 | 0.11085667 | 140370 |
| 11 | rs142920272 | rs17576165 | 0.889376 | 0.11085667 | 141991 |
| 11 | rs142920272 | rs17659953 | 0.889376 | 0.11085667 | 142115 |
| 11 | rs142920272 | rs34043286 | 0.889376 | 0.11085667 | 184721 |
| 11 | rs142920272 | rs36076725 | 0.889376 | 0.11083667 | 191308 |
| 11 | rs142920272 | rs7350928 | 0.889376 | 0.11081667 | 193740 |
| 11 | rs142920272 | rs17660464 | 0.889376 | 0.11076 | 123847 |
| 11 | rs142920272 | rs16940799 | 0.889376 | 0.11019 | 198907 |
| 11 | rs142920272 | rs1052551 | 0.889376 | 0.11018667 | 232916 |
| 11 | rs142920272 | rs17651549 | 0.889376 | 0.11018667 | 240562 |
| 11 | rs142920272 | rs1052553 | 0.884226 | 0.11255 | 227951 |
| 11 | rs142920272 | rs17652502 | 0.884226 | 0.11108333 | 207369 |
| 11 | rs142920272 | rs8712 | 0.884226 | 0.11030333 | 199969 |
| 11 | rs142920272 | rs9468 | 0.884226 | 0.11030333 | 200277 |
| 11 | rs142920272 | rs2668692 | 0.879132 | 0.11150333 | 8820 |
| 11 | rs142920272 | rs62073157 | 0.873891 | 0.11061667 | 32212 |
| 11 | rs142920272 | rs9303525 | 0.859103 | 0.18621667 | 114583 |
| 11 | rs142920272 | rs7221390 | 0.859103 | 0.18584333 | 184890 |
| 11 | rs142920272 | rs2066899 | 0.859103 | 0.18555333 | 146108 |
| 11 | rs142920272 | rs2732706 | 0.838497 | 0.10987667 | 49846 |
| 12 | rs55962189 | rs6035821 | 0.807819 | 0.34826 | 1132 |
| 13 | rs112635299 | rs28929474 | 0.883797 | 0.00680667 | 6805 |
| 14 | rs6701243 | rs35518820 | 0.810728 | 0.25807 | 56954 |
| 15 | rs45595836 | No Proxy |  |  |  |
| 16 | rs325485 | rs325481 | 0.90419 | 0.35383333 | 5384 |
| 16 | rs325485 | rs325502 | 0.841948 | 0.36851 | 12765 |
| 16 | rs325485 | rs254024 | 0.811341 | 0.42166 | 51348 |
| 16 | rs325485 | rs185260 | 0.811339 | 0.40071333 | 30145 |
| 16 | rs325485 | rs254020 | 0.806797 | 0.42117667 | 45117 |
| 17 | rs201910565 | No Proxy |  |  |  |
| 18 | rs210894 | No Proxy |  |  |  |
| 19 | rs72934503 | rs1487445 | 0.893466 | 0.35073667 | 18277 |
| 19 | rs72934503 | rs1906252 | 0.885746 | 0.35776667 | 33199 |
| 19 | rs72934503 | rs13208578 | 0.81953 | 0.36740333 | 10512 |
| 19 | rs72934503 | rs12204181 | 0.81953 | 0.37578 | 4007 |
| 20 | rs11185408 | rs12128610 | 0.952902 | 0.43638 | 2359 |
| 20 | rs11185408 | rs12406661 | 0.897413 | 0.40699 | 6508 |
| 21 | rs141455452 | No Proxy |  |  |  |
| 22 | rs78827416 | No Proxy |  |  |  |
| 23 | rs59566011 | No Proxy |  |  |  |
| 24 | rs10666089m | No Proxy |  |  |  |
| 25 | rs147317628 | No Proxy |  |  |  |
| 26 | rs292441 | rs292442 | 0.991021 | 0.45972 | 89 |
| 26 | rs292441 | rs506784 | 0.964285 | 0.32774 | 8592 |
| 26 | rs292441 | rs482805 | 0.947005 | 0.32939 | 8545 |
| 26 | rs292441 | rs2586982 | 0.947005 | 0.48966 | 4203 |
| 26 | rs292441 | rs497254 | 0.942749 | 0.47817333 | 6359 |
| 26 | rs292441 | rs495437 | 0.942446 | 0.46592 | 6447 |
| 26 | rs292441 | rs541427 | 0.918275 | 0.48484 | 1977 |
| 26 | rs292441 | rs292456 | 0.865926 | 0.48345 | 15621 |
| 26 | rs292441 | rs292443 | 0.855643 | 0.46646 | 624 |
| 26 | rs292441 | rs292453 | 0.854055 | 0.39181 | 15910 |
| 26 | rs292441 | rs292457 | 0.850131 | 0.42195 | 15432 |
| 26 | rs292441 | rs182383 | 0.813378 | 0.48917667 | 23197 |
| 26 | rs292441 | rs292451 | 0.802139 | 0.45197333 | 19753 |
| 27 | rs564835437 | No Proxy |  |  |  |
| 28 | rs4750990 | rs10741160 | 0.933777 | 0.35002333 | 2141 |
| 29 | rs117603308 | No Proxy |  |  |  |
| 30 | rs34938366 | No Proxy |  |  |  |
| 31 | rs35404050 | rs12829829 | 1 | 0.2181 | 4174 |
| 31 | rs35404050 | rs12422693 | 1 | 0.22715333 | 2173 |
| 31 | rs35404050 | rs7137061 | 1 | 0.22718 | 10914 |
| 31 | rs35404050 | rs11179386 | 0.988199 | 0.23504 | 18593 |
| 31 | rs35404050 | rs11179382 | 0.988199 | 0.25644 | 17255 |
| 32 | rs11480060 | rs1337241 | 0.820953 | 0.47250667 | 1910 |
| 32 | rs11480060 | rs2144623 | 0.816648 | 0.48336333 | 2601 |
| 32 | rs11480060 | rs1890051 | 0.811813 | 0.48228667 | 1642 |
| 32 | rs11480060 | rs1337242 | 0.811793 | 0.48848 | 5751 |
| 33 | rs740883 | rs29253 | 1 | 0.10776 | 5031 |
| 33 | rs740883 | rs29226 | 1 | 0.10759333 | 5606 |
| 33 | rs740883 | rs28359974 | 1 | 0.10759333 | 10279 |
| 33 | rs740883 | rs6919973 | 1 | 0.10745333 | 12469 |
| 33 | rs740883 | rs2021749 | 1 | 0.10724667 | 22714 |
| 33 | rs740883 | rs28359987 | 1 | 0.10481 | 19044 |
| 33 | rs740883 | rs29227 | 1 | 0.10480333 | 5183 |
| 33 | rs740883 | rs17184416 | 1 | 0.10474333 | 18073 |
| 33 | rs740883 | rs29258 | 1 | 0.10471333 | 2212 |
| 33 | rs740883 | rs29261 | 1 | 0.10470333 | 793 |
| 33 | rs740883 | rs29262 | 1 | 0.10470333 | 1187 |
| 33 | rs740883 | rs16867690 | 1 | 0.10470333 | 9978 |
| 33 | rs740883 | rs740881 | 1 | 0.10469333 | 162 |
| 33 | rs740883 | rs29263 | 1 | 0.10469333 | 1185 |
| 33 | rs740883 | rs28359967 | 1 | 0.10469333 | 4860 |
| 33 | rs740883 | rs29225 | 1 | 0.10469333 | 5636 |
| 33 | rs740883 | rs17854217 | 1 | 0.10469333 | 5705 |
| 33 | rs740883 | rs29257 | 1 | 0.10469 | 2625 |
| 33 | rs740883 | rs28359971 | 1 | 0.10469 | 9483 |
| 33 | rs740883 | rs28359975 | 1 | 0.10469 | 10777 |
| 33 | rs740883 | rs17842396 | 1 | 0.10469 | 10979 |
| 33 | rs740883 | rs29229 | 1 | 0.10468 | 453 |
| 33 | rs740883 | rs881284 | 1 | 0.10468 | 642 |
| 33 | rs740883 | rs28359983 | 1 | 0.10461 | 17274 |
| 33 | rs740883 | rs28359984 | 1 | 0.10461 | 17285 |
| 33 | rs740883 | rs17178014 | 1 | 0.10459667 | 17688 |
| 33 | rs740883 | rs740882 | 1 | 0.10458667 | 51 |
| 33 | rs740883 | rs29223 | 1 | 0.10450333 | 13139 |
| 33 | rs740883 | rs28359982 | 1 | 0.10443333 | 17240 |
| 33 | rs740883 | rs3025626 | 0.988707 | 0.10442667 | 16459 |
| 33 | rs740883 | rs29245 | 0.988707 | 0.10392667 | 15192 |
| 33 | rs740883 | rs29222 | 0.988707 | 0.10392667 | 15547 |
| 34 | rs16879023 | rs1150639 | 0.914228 | 0.23463333 | 9371 |
| 34 | rs16879023 | rs1144696 | 0.885468 | 0.23560333 | 10222 |
| 34 | rs16879023 | rs2301594 | 0.817552 | 0.26870667 | 1219 |
| 34 | rs16879023 | rs1322599 | 0.806661 | 0.28776 | 5278 |
| 35 | rs141319505 | No Proxy |  |  |  |
| 36 | rs77691144 | No Proxy |  |  |  |
| 37 | rs4916723 | rs17422060 | 0.835088 | 0.29777667 | 4535 |
| 38 | rs2635182 | No Proxy |  |  |  |
| 39 | rs10110094 | No Proxy |  |  |  |
| 40 | rs12942300 | No Proxy |  |  |  |
| 41 | rs78058104 | rs114941847 | 1 | 0.04178 | 2591 |
| 41 | rs78058104 | rs8042369 | 0.969591 | 0.04349 | 4161 |
| 42 | rs1480899431 | No Proxy |  |  |  |
| 43 | rs28729902 | rs12339193 | 0.943618 | 0.12977333 | 6429 |
| 43 | rs28729902 | rs78640976 | 0.913131 | 0.08653 | 9379 |
| 43 | rs28729902 | rs1497027 | 0.913131 | 0.08571 | 16030 |
| 43 | rs28729902 | rs12351872 | 0.912438 | 0.13350333 | 12924 |
| 43 | rs28729902 | rs73503170 | 0.912438 | 0.13317667 | 11310 |
| 43 | rs28729902 | rs17658759 | 0.864805 | 0.08145333 | 12926 |
| 43 | rs28729902 | rs77495304 | 0.863676 | 0.14918667 | 65331 |
| 43 | rs28729902 | rs77084713 | 0.857479 | 0.15567333 | 86347 |
| 43 | rs28729902 | rs993350 | 0.851789 | 0.08107333 | 53190 |
| 43 | rs28729902 | rs10512021 | 0.821449 | 0.08044667 | 150919 |
| 44 | rs144911765 | No Proxy |  |  |  |
| 45 | rs11787216 | rs1078141 | 0.890465 | 0.22311 | 4171 |
| 45 | rs11787216 | rs13266268 | 0.830429 | 0.22359333 | 3251 |
| 45 | rs11787216 | rs11782665 | 0.825866 | 0.26981667 | 11249 |
| 46 | rs33966416 | rs1522603 | 0.980213 | 0.44234 | 433 |
| 46 | rs33966416 | rs7657061 | 0.968509 | 0.44413333 | 8828 |
| 46 | rs33966416 | rs4692812 | 0.893273 | 0.47052 | 26330 |
| 47 | rs200332011 | No Proxy |  |  |  |
| 48 | rs7000276 | rs7821914 | 0.837929 | 0.44023 | 172 |
| 49 | rs13188074 | No Proxy |  |  |  |
| 50 | rs9389208 | rs9376056 | 0.949622 | 0.36747333 | 8767 |
| 51 | rs6692705 | rs10494688 | 0.991658 | 0.42524333 | 1396 |
| 51 | rs6692705 | rs1555649 | 0.975322 | 0.41136 | 25564 |
| 51 | rs6692705 | rs59097021 | 0.962961 | 0.48359667 | 1925 |
| 51 | rs6692705 | rs1408408 | 0.950324 | 0.49019 | 8992 |
| 51 | rs6692705 | rs7554157 | 0.950324 | 0.49038333 | 13440 |
| 51 | rs6692705 | rs10921376 | 0.83586 | 0.3557 | 12496 |
| 51 | rs6692705 | rs927999 | 0.813776 | 0.35587667 | 2926 |
| 52 | rs76397219 | No Proxy |  |  |  |
| 53 | rs888972579 | No Proxy |  |  |  |
| 54 | rs1312643832 | No Proxy |  |  |  |
| 55 | rs79940520 | No Proxy |  |  |  |
| 56 | rs201369005 | No Proxy |  |  |  |
| 57 | rs78653484 | No Proxy |  |  |  |
| 58 | rs12203328 | rs12211578 | 0.989267 | 0.16254 | 45088 |
| 58 | rs12203328 | rs72835585 | 0.983928 | 0.16360667 | 5402 |
| 58 | rs12203328 | rs9393479 | 0.978821 | 0.25442333 | 23174 |
| 58 | rs12203328 | rs9366536 | 0.978644 | 0.19427333 | 33348 |
| 58 | rs12203328 | rs9348604 | 0.978644 | 0.19208333 | 31257 |
| 58 | rs12203328 | rs9379588 | 0.978644 | 0.19199667 | 29140 |
| 58 | rs12203328 | rs9356902 | 0.978644 | 0.19190333 | 29318 |
| 58 | rs12203328 | rs9379577 | 0.973632 | 0.18364 | 8557 |
| 58 | rs12203328 | rs9356900 | 0.973402 | 0.25654667 | 26001 |
| 58 | rs12203328 | rs36086996 | 0.968202 | 0.18130667 | 7385 |
| 58 | rs12203328 | rs12213691 | 0.968202 | 0.16875333 | 37183 |
| 58 | rs12203328 | rs13206774 | 0.914435 | 0.21597 | 202 |
| 59 | rs116977567 | rs150327164 | 0.922523 | 0.01745 | 17960 |
| 60 | rs529507 | rs492768 | 0.937339 | 0.15674667 | 8838 |
| 60 | rs529507 | rs523871 | 0.877719 | 0.16562 | 9938 |
| 60 | rs529507 | rs35920710 | 0.833423 | 0.15388667 | 11134 |
| 61 | rs77810738 | rs117173030 | 0.971221 | 0.02501 | 45652 |
| 62 | rs113003385 | No Proxy |  |  |  |
| 63 | rs12449792 | No Proxy |  |  |  |
| 64 | rs6430841 | rs9646701 | 0.915548 | 0.16077333 | 118581 |
| 64 | rs6430841 | rs7597242 | 0.869405 | 0.10949667 | 65809 |
| 65 | rs13201465 |  |  |  |  |
| 66 | rs7578456 | rs2349075 | 0.963351 | 0.31431667 | 11782 |
| 66 | rs7578456 | rs12052257 | 0.959345 | 0.36664 | 6246 |
| 66 | rs7578456 | rs6705845 | 0.932569 | 0.48078333 | 16309 |
| 66 | rs7578456 | rs6710974 | 0.932569 | 0.48116667 | 21966 |
| 66 | rs7578456 | rs12472273 | 0.913758 | 0.49161 | 23653 |
| 66 | rs7578456 | rs1406122 | 0.913758 | 0.49230667 | 23905 |
| 66 | rs7578456 | rs10202963 | 0.897747 | 0.47094333 | 6559 |
| 66 | rs7578456 | rs9288318 | 0.894073 | 0.32327667 | 39285 |
| 67 | rs34739626 | No Proxy |  |  |  |
| 68 | rs6082289 | rs2064819 | 0.911424 | 0.16802333 | 3922 |
| 68 | rs6082289 | rs4599194 | 0.911424 | 0.16652667 | 2026 |
| 69 | rs597040 | No Proxy |  |  |  |
| 70 | rs148587110 | No Proxy |  |  |  |
| 71 | chr6:96509180 | No Proxy |  |  |  |
| 72 | rs62256326 | No Proxy |  |  |  |
| 73 | rs116346488 | No Proxy |  |  |  |
| 74 | rs507059 | No Proxy |  |  |  |
| 75 | rs2282828 | No Proxy |  |  |  |
| 76 | rs143609523 | No Proxy |  |  |  |
| 77 | rs56054767 | No Proxy |  |  |  |
| 78 | rs41363353 | rs11901070 | 0.924778 | 0.13448333 | 54352 |
| 78 | rs41363353 | rs78995299 | 0.862311 | 0.07899667 | 34466 |
| 78 | rs41363353 | rs67789268 | 0.814126 | 0.13966667 | 134125 |
| 78 | rs41363353 | rs6744226 | 0.809213 | 0.13929333 | 154116 |
| 79 | rs115833252 | No Proxy |  |  |  |
| 80 | rs78298487 | rs73724045 | 1 | 0.06913 | 1742 |
| 80 | rs78298487 | rs156928 | 0.838278 | 0.12906667 | 4921 |
| 81 | rs4609618 | rs7938490 | 0.934265 | 0.47567667 | 10792 |
| 82 | rs16933101 | rs7307357 | 0.892453 | 0.03591 | 134636 |
| 83 | rs113764414 | No Proxy |  |  |  |
| 84 | rs17517971 | No Proxy |  |  |  |
| 85 | rs684502 | No Proxy |  |  |  |
| 86 | rs34509057 | No Proxy |  |  |  |
| 87 | rs201179706 | No Proxy |  |  |  |
| 88 | rs71868489 | No Proxy |  |  |  |
