# Supplementary material for: Commonly used genomic arrays may lose information due to imperfect coverage of discovered variants for autism spectrum disorder
Source: J Neurodev Disord. 2024 Sep 12;16:54. doi: 10.1186/s11689-024-09571-8 (PMC11397030; doi:10.1186/s11689-024-09571-8)
Supplement: Supplementary file 3 — Additional file 3: Supplementary Table 3. Characteristics of ASD-PGS Derivation in Reported Studies. Contains the results of the literature search on characteristics of ASD-PGS derivation in 24 studies, including information from the following categories: ASD Discovery GWAS, Target GWA Chip, Imputed/Reference Panel, Post-imputation filters, PRS software, Clumping reference panel, Clump r2, clump window size, presence of a 2nd clump round, specification of a PRS threshold, and whether # of SNPs was reported. [file 11689_2024_9571_MOESM3_ESM.docx]

| **Supplementary Table 3. Characteristics of ASD-PGS Derivation in Reported Studies** | | | | | | | | | | | | |
| --- | --- | --- | --- | --- | --- | --- | --- | --- | --- | --- | --- | --- |
| **PMID** | **ASD Discovery**  **GWAS** | **Target GWA Chip** | **Imputed/**  **Reference Panel** | **Post-Imputation Filters** | | **PRS Software** | **Clumping Reference Panel** | **Clump r^2^** | **Clump Window Size (kb)** | **2nd Clump Round?** | **PRS Threshold Specified?** | **# SNPs reported?** |
| 31346826 | Grove | Ipsych | No mention so assume measured genotypes | No mention | | PRSice | No mention | 0.1 | 250 | No mention | No mention | Yes in Supp |
| 31571410 | Grove | Infinium OmniExpress24v1.1 (~700k) | MACH using 1000g European samples | INFO>.8 | | PLINK | 1000g European | 0.2 | 500 | No mention | yes | The scores included contributions from about 32,000 variants |
| 29898212 | PGC (5305) | Illumina HumanHap 550  Quad | IMPUTE2; 1000g all ancestries (n=2186) | INFO>.8 | | PLINK | No mention | 0.25 | 500 | No mention | No mention | No mention |
| 34615521 | Grove | Omni 1M, 2.5M, 660 Quad, Exome8 | Michigan Server; HRC | Yes; criteria not specified | | PLINK | No mention | 0.8 | 50 | yes | 0.01; optimized? agree | Yes in Supp |
| 31659270 | Grove | Applied Biosystems UK Biobank Axiom Array, Applied Biosystems UK BiLEVE Axiom Array by Affymetrix | Imputed described in other UK biobank paper - used best guess calls  HRC, merged UK10K and 1000 Genomes phase 3 reference panels, using IMPUTE4 | r2>0.6 | | PRSice2 | No mention | 0.2 | 250 | No mention | No mention | Yes in Supp |
| 28044064 | PGC-ASD (info>.8); PGC-SZ2 (info>.9) | Illumina 550 | iMPUTEv2.2; best guess call; 1000g supp doesn't say euro only or provide N | INFO>.8 | | PLINK | 1000g (no N or mention of European only) | 0.25 | 500 | No mention | No mention | No mention |
| 28507316 | PGC-ASD | 370CNV Beadchip; Omni | IMPUTE2 | INFO ⩾ 0.6 | | PRSice | No mention | 0.25 | 500 | No mention | No mention | No mention |
| 35228676 | Grove | ALSPAC: Illumina Human Hap 550; TEDS: Affy 6.0 or OmniExome3v1.2 | no mention, but likely in other papers; HRC ref | No mention | | PLINK | No mention | 0.25 | 500 | No mention | No mention | Yes in Supp |
| 32031653 | Grove | Japonica array ~650k | beagle 5.0; 1000g | INFO>.8 | | PRSice1 | No mention | 0.1 | 1000 | No mention | Optimized at p=?? | ~5.6 M snps in score; T2 gives optimized thresh and N snps |
| 31629460 | PGC-ASD | Illumina 610 or 660 | shapeit; impute; 1000g ref | INFO>.9 | | PRSice | No mention | 0.1 | 250 | No mention | No mention | Yes in Supp |
| 25754080 | PGC-ASD (5314) | Illumina OmniExpress Exome8 (~1M inclding .25M exonic) | not for Lothian and Generation Scotland; only Brisbane; 1000g ref | R2>.3 for Brisbane only | | PLINK | No mention | 0.25 | 300 | No mention | p=.5 | No mention |
| 32954337 | Grove | Illumina 660 | Sanger impuation service; HRC | INFO>.9; maf>0.05 | | PRSice2 | No mention | 0.1 | 250 | No mention | Only considered p=.001 | Yes |
| 33229037 | PGC-ASD minus SSC samples | Omni 2.5 or 1M | via Mich Server; 1000g in supp but HRC in paper; best guess calls | r2>.3 | | PRScs-auto and PLINK | CEU panel | No mention | No mention | No mention | No mention | only a single score to evaluate from Bayesian approach using 1,113,041 SNPs |
| 28504703 | ipsych first 10 waves(7,783 cases and 11,359 controls) | Omni 2.5 or 1M | details in SZ Workshop supp; 1000g ref | INFO>.6 | | PLINK? | 1000g according to Ricopilli pipeline | 0.1 | 500 | No mention | Optimized at p=.10 | Yes |
| 9418275 | PGC-ASD |  | shapeit; impute2; EUR 1000g ref | Zhou et al | | Plink v1.9 | Maybe Ding et al? | No mention | No mention | No mention | No mention | No mention |
| 32624584 | PGC-ASD | Illumina Human Omni Express 12v1-H chips(DE-Cohort) 550 K Illumina, 510 K Illumina, 1 M Single, and 1 M Duo Illumina chips(AGP samples), 1 M Illumina | “Based on” Minimac3(maybe Beagle?); 1000g ref | Rsq>0.3, rerun QC pipeline | | PRSice | No mention | No mention | No mention | No mention | No mention | 6,900,500 SNPs |
| 34856637 | Grove | Illumina HumanHap550 quad chip, Illumina Human660W-quad array(ALSPAC) | “multiple imputation (n = 20) with chained equations following the ‘H0’ approach” | No mention | | PRSice | No mention | No mention | No mention | No mention | “p=0.1 has best predictive power in children” | No mention |
| 30566181 | Independent discovery data | Illumina PsychChip | CATSS | Brikell et al | | PLINK v1.9 | No mention | No mention | No mention | No mention | P<.50 | 6 981 993 imputed SNPs |
| 34605010 | Grove | Illumina HumaneCoreExome, Illumina Global Screening Array | HRC, Sanger Imputation Server | genotyping call rate greater than 99%, European 2 ethnicity, INFO>0.8, MAF>0.01, HW Equilibrium P>10^-6 | | PRSice2 | No mention | No mention | No mention | No mention | Yes, <5e−8, <1e−6, <1e−4, <.001, <.01, <.05, <.1., <.2, <.5. and 1 | Yes |
| 33568206 | Grove | Illumina Global Screening Array v1 and v2 | Sanger Imputation Service & EAGLE2/ Haplotype Reference Consortium | HWE p<1e-6, MAF <0.01, info <0.8 | | SBayesR+ PLINK | No mention | No mention | No mention | No mention | No mention | No mention |
| 34615521 | Grove | Human1M_v1, Human1M_Duov3, and HumanOmni-2.5 for SSC; Infinium OmniExpressExome-8 V1, Infinium OmniExpressExome-8 V1.1-V1.4, and Infinium Expanded Multi-Ethnic Genotyping Array, Illumina Human660W_Quad_v1_A | Michigan Imputation Server, HRC Ref | No mention | | No mention | No mention | 0.81 | 50 kb | No mention | 0.01 | Yes |
| 29660215 | PGC-ASD | Illumina Human660W-Quad BeadChip | MaCH, minimac, 1000g ref | R quality>0.3, MAF>0.01, per subject missingness<.01, autosomal, unambiguous strand alignment | PLINK 1.9 | | No mention | 0.1 | 500 kb | No mention | Yes <.05 | Yes |
| 35654974 | Grove | 3 different cohorts. REACH,  Simons Simplex, SPARK (Illumina global screening array GSA-24v1-0) | RICOPILI pipeline | No mention | SbayesR + PLINK (PRSice v2.3.0 as well but not used in paper) | | No mention | Mentioned but not used in paper | Mentioned but not used in paper | Mentioned but not used in paper | Mentioned but not used in paper | No mention |
| 36028495 | Gui | SmokeScreen Genotyping Array | Impute to HapMap3 SNPs in the full 1000G panel | Info >0.8 | SDPR | | No mention | No mention | No mention | No mention | No mention | Yes |
