# Supplementary material for: Commonly used genomic arrays may lose information due to imperfect coverage of discovered variants for autism spectrum disorder
Source: J Neurodev Disord. 2024 Sep 12;16:54. doi: 10.1186/s11689-024-09571-8 (PMC11397030; doi:10.1186/s11689-024-09571-8)
Supplement: Supplementary file 4 — Additional file 4: Supplementary Table 4. PGS coverage metric by Discovery P-value threshold. Contains values of our novel PGS coverage metric for IBIS, EARLI, MARBLES, and SEED for 9 selected P-values. [file 11689_2024_9571_MOESM4_ESM.docx]

| **Supplementary Table 4. PGS coverage metric by Discovery P-value threshold** | | | | | | | | | | |
| --- | --- | --- | --- | --- | --- | --- | --- | --- | --- | --- |
| **Cohort** | **5.00E-08** | **1.00E-06** | **1.00E-04** | **0.001** | **0.01** | **0.05** | **0.1** | **0.2** | **0.5** | **1** |
| **IBIS** | 1 | 0.921 | 0.9806 | 0.9668 | 0.9711 | 0.9657 | 0.964 | 0.9618 | 0.9572 | 0.9569 |
| **EARLI** | 1 | 0.921 | 0.9805 | 0.9625 | 0.964 | 0.9557 | 0.9543 | 0.9506 | 0.9454 | 0.9445 |
| **MARBLES** | 1 | 0.7989 | 0.9456 | 0.9354 | 0.9426 | 0.94 | 0.9392 | 0.9351 | 0.9314 | 0.9295 |
| **SEED** | 1 | 0.9695 | 0.9686 | 0.9679 | 0.9778 | 0.9814 | 0.984 | 0.9841 | 0.9852 | 0.9849 |
